# Historic review: the foundation-period and the first 15 years of the “Gesellschaft fuer Neuropaediatrie” (GNP) between 1950 and 1990

**DOI:** 10.1186/s42466-020-00067-z

**Published:** 2020-10-06

**Authors:** Hans Michael Strassburg

**Affiliations:** 1 Gerbrunn, Germany; 2 Formerly Universitäts-Kinderklinik Würzburg, Josef Schneiderstr. 2, 97080 Würzburg, Germany

**Keywords:** Gesellschaft fuer Neuropaediatrie, Foundation of the Neuropediatric Society of German-speaking countries, 1950 till 1990 - organization structures - scientific projects - international contacts

## Abstract

After the end of Nazi terrorism with many crimes against neuropsychiatric and disabled people, from 1950 on detection and treatment of these diseases in children and adolescents grew continuously: in the 50s and 60s, several German books on neuropediatric topics were published, a journal was founded, and junior pediatricians established many scientific contacts with foreign neuropediatricians. In 1972, the team of the Kehl-Kork Epilepsy Clinic invited neuropediatric colleagues from Germany, Austria, and Switzerland for a first joint workshop. On March 4th, 1975, the Neuropaediatric Society of the German-speaking countries was founded in Heidelberg and its goals, as well as guidelines for membership, were formulated. The first annual meeting took place 1975 in Heidelberg as well. Between 1975 and 1990, the number of members of the GNP continued to increase from 140 to more than 230, despite strict admission criteria. The president was elected for a one-year term and was responsible for organizing the annual meeting, which took place alternately in Germany, Austria or Switzerland. The continuity of the society was ensured by secretary and treasurer as well as several longtime assessors. The meetings covered the entire spectrum of neurological and neuropsychological disorders in children. In addition to the age-dependent clinical investigation, the most important diagnostic methods were electrophysiology, in particular, the EEG and EMG, the new possibilities of cerebral imaging utilizing X-ray computed tomography and ultrasound, and biochemical analyzes for detection of metabolic diseases. Research projects were mostly carried out in university institutions and were only partially multi-center or financed with public funds. International contacts took place on many levels, e.g. through scholarships and personal exchanges with European and US scientific societies and institutions. Unfortunately, the opportunity to exchange ideas with colleagues from the German Democratic Republic (GDR) was limited. Several working groups addressed controversial issues of developmental neurology, epileptology, and alternative therapies. With the establishment of social pediatric centers in Germany from the late 1980s, there was an increasing demand for well-educated and experienced neuropediatricians.

## Background

Since the fifties of the twentieth century, pediatrics has undergone a tremendous transition to increasing specialization. One of the most important clinical areas are the neurological and functional diseases in children and adolescents. Their development between 1950 and 1990 in Germany will be presented below [24–26].

## Introduction

After the end of the Second World War and the atrocities of the Nazi era especially in dealing with neurologically ill and disabled children and adolescents, in the first years improving of the living conditions, especially overcoming the famine, the reduction of infant mortality and treatment of acute infectious diseases, e.g. tuberculosis and poliomyelitis, were in the foreground of the pediatricians interest. In the calling for chairs and head offices of pediatric hospitals, only a few applicants were found who were not involved in the machinations of the National Socialists (e.g. Bremen, Hamburg, Erlangen). Often, pediatricians were installed who had managed somehow to be grouped as “followers” despite still following fascistic ideas, to regain leading positions, such as in Goettingen, Berlin, and Munster. A quite blatantly example was the former chief expert of the “ Reichsausschuss zur wissenschaftlichen Erfassung erb- und anlagebedingter schwerer Leiden” (Committee for the scientific recording of genetic heredity and system-related severe suffering) and director of the Leipzig University Children’s Hospital Werner Catel, who was appointed to the University Children’s Hospital in Kiel in 1954 and continued there to promote his ideology of killing “unworthy life” [1, 3, 4, 26]. The shocking report on the first Nuremberg medical trials in “Medicine without Humanity” by Alexander Mitscherlich and Fred Mielke, published for the first time in 1947, was primarily passed on to members of the medical associations and was largely neglected; one could not and did not want to believe in public that physicians were involved in the abuses and killings of so many thousands of people, including many children [14]. It took decades for an objective public discussion of the past to be possible in Germany.

### Neuropediatric topics at the annual conferences of the German Society of Paediatrics (DGfK)

In 1950, at the annual meeting of the German Society of Pediatrics (Deutsche Gesellschaft fuer Kinderheilkunde DGfK) in Lübeck, a main topic was the “psychopathology of childhood”. The psychiatrist and neurologist W. Villinger (Marburg), who had been burdened by the Nazi period, gave a lecture on “Abnormal emotional reactions in childhood” and H. Asperger (Vienna) on “The medical basis of special education”. 1951 in Heidelberg, the electroencephalography was discussed, e.g. with reviews of the neurologist R. Jung from Freiburg and the pediatrician H. Pache from Munich. 1953 at the meeting in Bad Kissingen among others A. Matthes, Heidelberg, and H. Gött, Bonn reported on “infantile spasms”, also in Heidelberg in 1961 under the chairmanship of P. Bamberger, main topics were cerebral seizures and congenital metabolic anomalies. Interesting aspects in neuropediatrics in the following years were e.g.1966 a lecture by J. Paul (Erlangen) on the “mistreatment of brain-damaged children” and several lectures on the so-called “brain atrophic process”, e.g. dementia in childhood, which was beginning to be differentiated. 1967 in Vienna “Early detection and early treatment of neurological sensory and mental defects”, 1970 in Marburg “human genetic problems of pediatrics” and 1972 in Bad Pyrmont “The innate brain damage” were presented. 1973 in Nuremberg were “The inflammatory diseases of the central nervous system”, 1976 in Cologne “Normal and disturbed brain development - early diagnosis of cerebral damage” and “Psychopathology of minor brain dysfunction” were main topics, in Kiel 1977 “Syndromes - meaning and significance for clinic and practice “ [24, 30].

### Increasing interest in diseases of the nervous system in children

On September 26, 1951, the “German Association for Adolescent Psychiatry” (Deutsche Vereinigung fuer Jugendpsychiatrie DVJ) was founded in Stuttgart under the chairmanship of Werner Villinger, already mentioned before. The inclusion of pediatricians was initially rejected because of their “numerical superiority” (!). The first co-opted member of the board of DVJ was the pediatrician Carl Gottlieb Bennholdt-Thomsen from Cologne. In 1968, with the support of the DGfK, the specialty “child and adolescent psychiatry” was officially recognized [4, 24, 26, 27]. In 1957, the German Society for Epileptology (former Epilepsy League) was founded as a section of the International League Against Epilepsy (ILAE). In 1958, the founding of the “Bundesvereinigung Lebenshilfe” for the mentally handicapped child took place in Marburg through the initiative of the Dutchman Tom Mutters (1917–2016) and concerned parents to improve the inadequate situation of caring for intellectually impaired children and adolescents. In more and more cities early childhood care centers, special kindergartens and special schools were created starting from the beginning of the sixties with support of churches and charitable organizations as well as some engaged politicians [24, 25].

### Milestones of clinical-scientific neuropediatrics until 1970

**Fig. 1:**
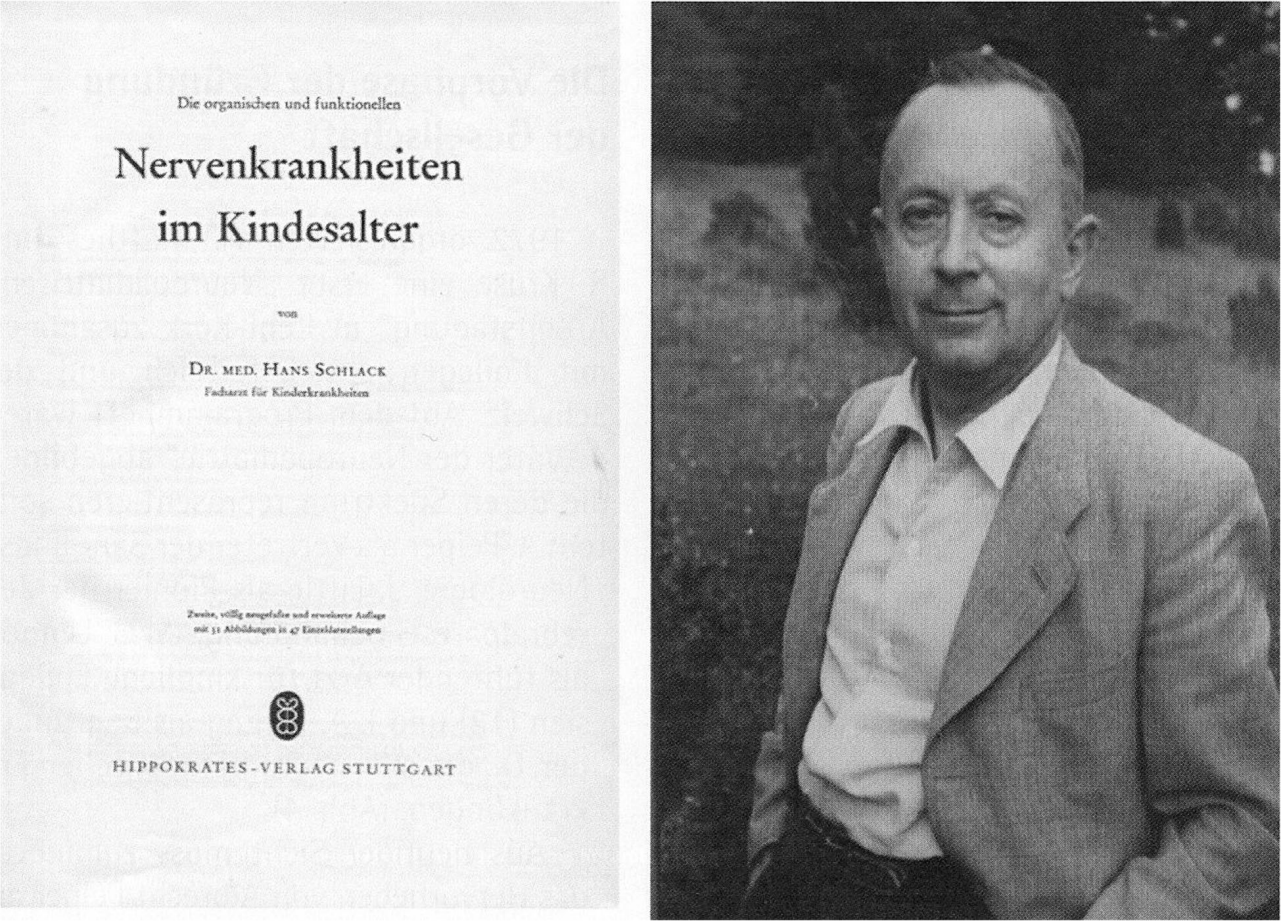
**a** and **b** The first page of the monography „Die organischen und funktionellen Nervenkrankheiten im Kindesalter” by Hans Schlack (1898–1971), published 1961 and its author [21]

In 1949, the monograph on “Die Eigenart der kindlichen Hirntätigkeit” by Albrecht Peiper, (Greifswald and Leipzig, 1889–1968) appeared with various registrations of body movements, respiration and behavior of infants and toddlers [17]. Less well known is the first edition of a German practice-oriented book after the 2nd World War, “Die organischen und funktionellen Nervenkrankheiten des Kindesalters” by the Stuttgart-based pediatrician Hans Schlack (1898–1971). He mainly describes his extensive clinical experience in children with infectious diseases of the CNS, in particular poliomyelitis, but also in detail congenital malformations, epilepsy and functional disorders. As principles Schlack formulates “as little as possible to remove the child from the domestic milieu” and “treat the impossible as if it were possible” (Goethe) [21]. Fig. 1.

**Fig. 2:**
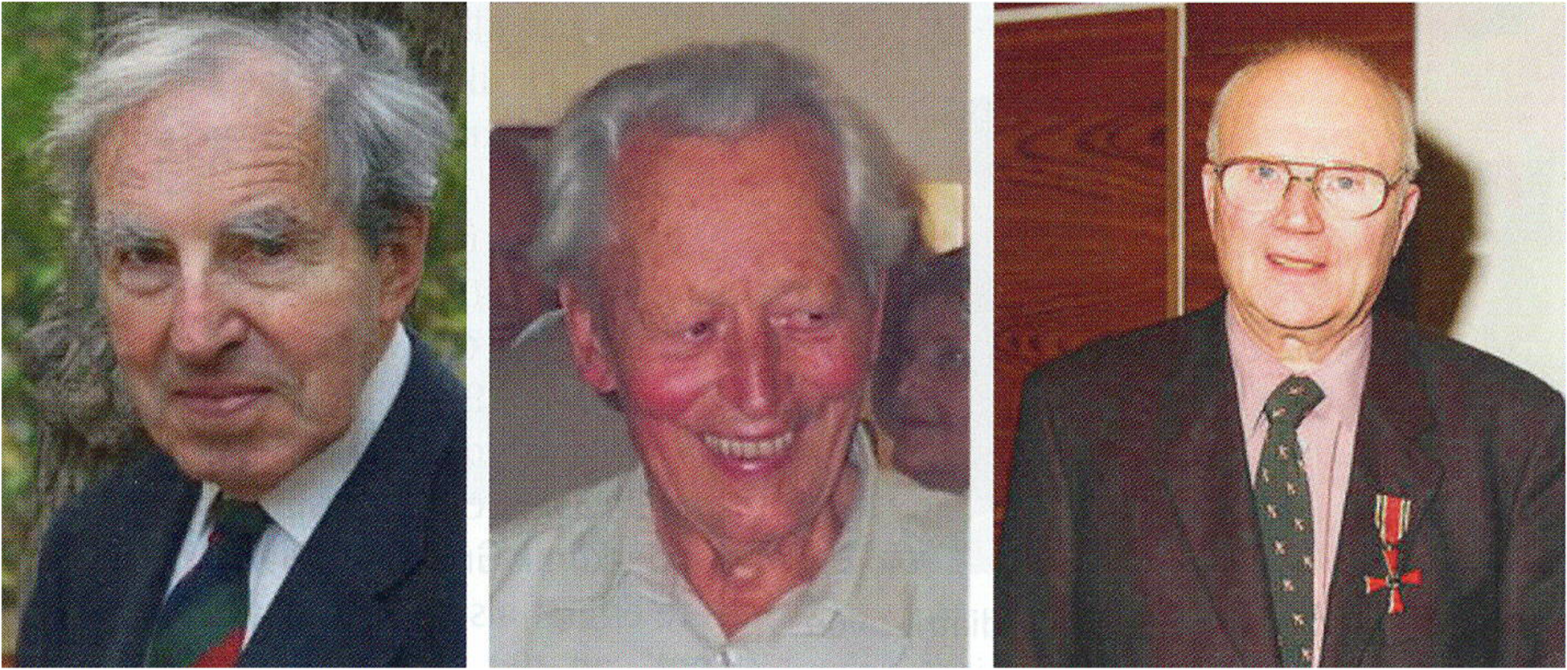
**a**,**b** and **c** Dieter Janz, Ansgar Matthes, and Rolf Kruse, precursors of neuropediatrics in Germany

At the University Children’s Hospital in Heidelberg, there was great interest in neurological diseases of children, especially epileptology, under Philipp Bamberger, and the neurologists Paul Vogel (1900–1979) and Dieter Janz (1920–2016). In 1959 Ansgar Matthes (1924–2008) published together with Bamberger the monograph “Seizures in Childhood”. 1966 Rolf Kruse (1928–2010) habilitated with a study on the myoclonic-astatic petit mal [2, 23] (Fig. 2a-c).

A new era began in 1967 when Professor A. Matthes from Heidelberg founded an outpatient clinic for children with epilepsy in the Korker Anstalten near Kehl at the Rhein. In 1969, Prof. Kruse from Heidelberger joined him and together with Matthes directed the specialized clinic for children with seizures.

In Göttingen there was also a very active group of scientists in the hospital as well as in theoretical institutes. In 1968, the extensive monograph on “Neurology of the Newborn” by Gerhard Joppich and Franz Joseph Schulte with many clinical and pathoanatomical findings, neurophysiological registrations and the still impressive number of 4342 references (!) appeared [9].

F.J. Schulte and Hans Gerd Lenard cultivated a variety of international contacts with neuropediatrically active colleagues, e.g. to the USA, the UK, Sweden, and Switzerland. In 1972, at the Children’s University Hospital Göttingen, the first professorship for pediatrics with a focus on neuropediatrics was set up in Germany and F.J. Schulte got this position. In the same year, after more than 3 years of training in the USA, Joest Martinius completed his academic career at the Max Planck Institute in Munich and for the first time became a lecturer for “neuropediatrics”.

1973 appeared the extensive monograph “Neuropädiatrie”, edited by A. Matthes and R. Kruse. Among 18 authors were Helmuth Remschmidt and Peter Strunk as child and adolescent psychiatrists. According to the foreword, 3 main topics should be addressed: common neuropediatric diseases, socio-medical important neuropediatric diseases, and neurometabolic diseases. Particular emphasis was put on the neuropediatric examination methods, especially in neonates, and the integration of psychological findings into the assessment of patients [12].

Since the beginning of the sixties the Austrian Heinz F.R. Prechtl, a student of Konrad Lorenz, and his research group in Groningen published groundbreaking investigations in the young infant and its behavior, thus influencing German pediatricians [19].

Together with the Würzburg (originally Göttingen) neurosurgeon Karl August Bushe (1921–1999), F. J. Schulte founded the journal “Neuropädiatrie” which was changed from 1980 to the English language “Neuropediatrics - Journal of Pediatric Neurobiology, Neurology and Neurogenetics”.

### The preliminary phase of the founding of the society

**Fig. 3:**
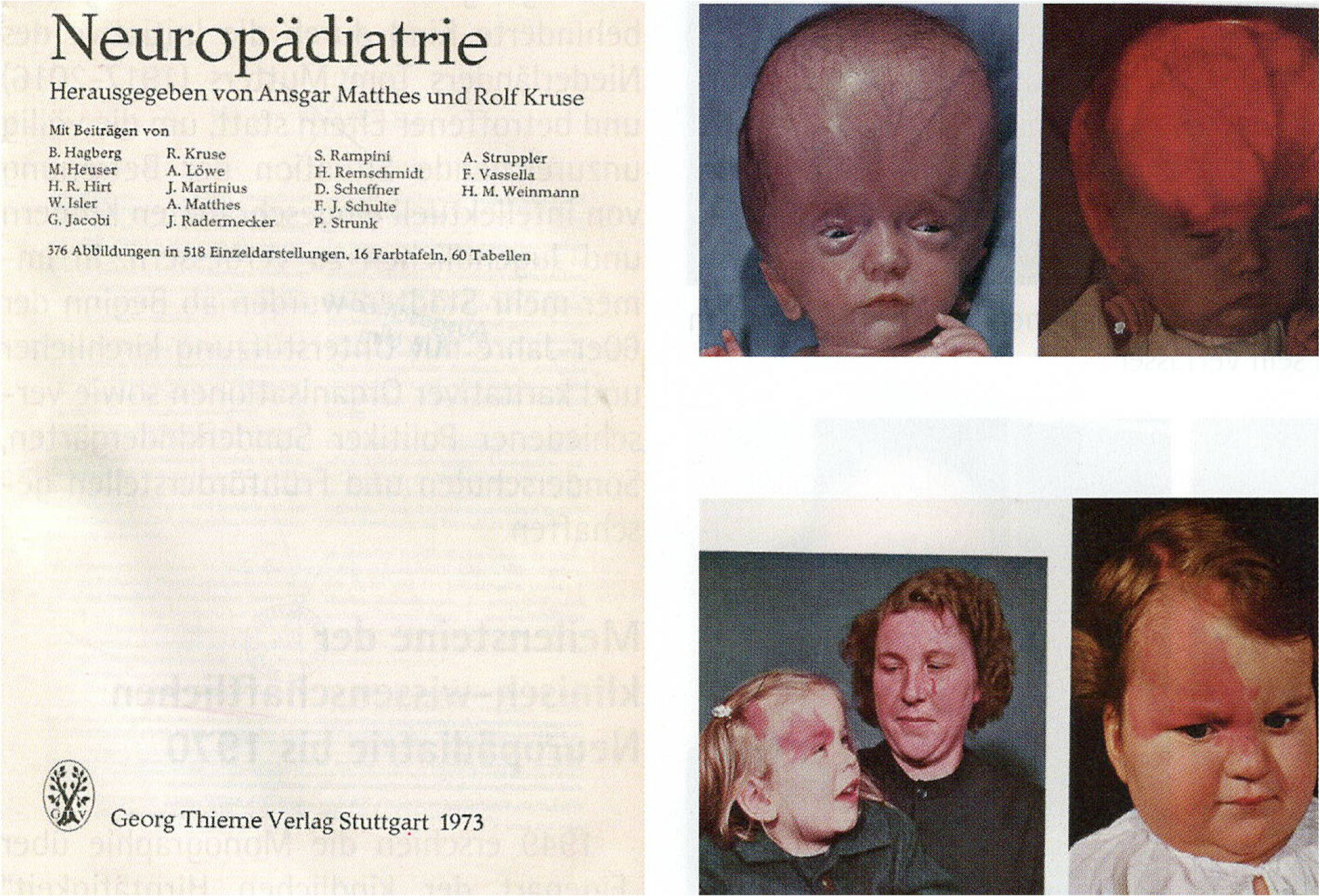
The monography "Neuropädiatrie” of 1973 edited by A. Matthes and R. Kruse with figures of a child with an extreme hydrocephalus and its diaphanoscopy and of a child with Sturge-Weber-syndrome [12]

In 1972, A. Matthes and R. Kruse organized a first “Neuropediatric Workshop” in Kehl-Kork together with colleagues from Austria and Switzerland. The program booklet depicted four “fathers of neuropediatrics” who should show the spectrum: Albrecht Peiper as a representative of infant neurology, William John Little as a pioneer of cerebral palsy, William Gordon Lennox as the leading physician in pediatric epilepsy and Ivar Asbjoern Fölling as the founder of diagnosing of neurometabolic diseases Fig. 3.

**Fig. 4:**
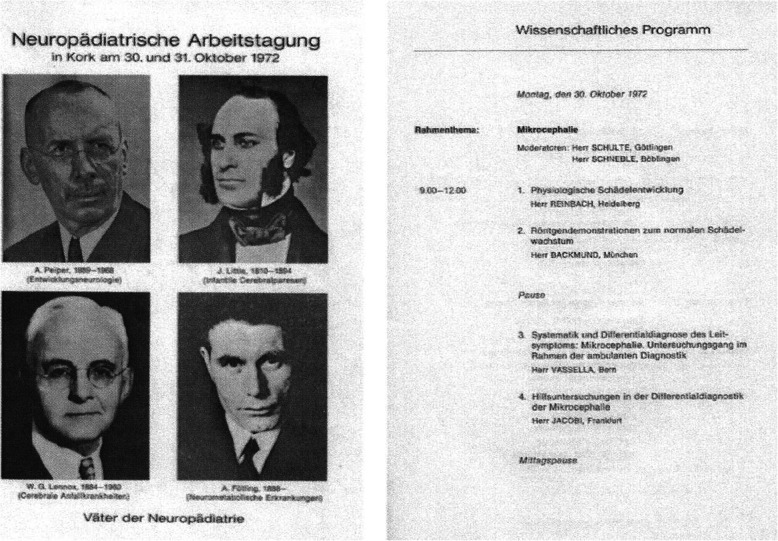
The first pages of the Neuropediatic workshop program in Kehl-Kork 1972

From today’s point of view, at least the highlighting of A. Peiper must be considered as problematic: in his work “Die Mitarbeit des Kinderarztes an der Artverbesserung”, published in 1934 in KINDERÄRZTLICHE PRAXIS, there are many defamatory passages to so-called “ill” children, the “inferiority” of artificially fed infants, and an exuberant praise for the “new policy” [16, 29]. In 1937 he joined the NSDAP and in 1943 became chairman and deputy director of the Children’s University Hospital Greifwald, from 1948 to 1958 he was full professor of pediatrics at the Children’s University Hospital Leipzig. He has earned undeniable merits through his research on pediatrics history, which has been published as “Chronik der Kinderheilkunde” [17, 18, 24]. Fig. 4.

From December 7th to 9th 1973 a seminar for neuropediatric training and research took place in Göttingen, which was organized by F.J. Schulte and H.G. Lenard. There were reviews on the following topics: systemic muscle diseases presented by Victor Dubowitz, neurological complications in cyanotic heart diseases, endocrine diseases and the CNS and intranatal encephalopathies.

Between November 29th and December 1st, 1974 Franco Vasella addressed the “3. Jahrestagung der Arbeitsgemeinschaft Neuropaediatrie “ in Bern/Switzerland. Topics were progressive brain diseases, neurometabolic disorders, and spinal cord lesions.

### The foundation of the GNP

Thus, especially from the sixties of the twentieth century, there were increasing international and national activities in the field of neurological diseases in children, so that the continuously increasing amount of knowledge deserved specialization. Accordingly, there was a lot of discussion about founding a separate scientific society for neuropediatrics as part of pediatrics, e.g. at informal meetings in the guesthouse of the Max Planck Institute Munich between 1973 and 1975. Since 1973, the German Society for Pediatric Cardiology was the first sub-specialty, which emerged from a “Working Group for Pediatric Cardiology”, founded on 14.10.1969.

Again, pediatricians were criticized by representatives of neurology, orthopedics or physiotherapy, to have not enough skills for the classical neurological diseases, e.g. for the diagnosis and treatment of epilepsy and cerebral palsy, diseases of the muscles and peripheral nerves, as well as strokes or headaches. Finally, the delineation of competencies at the interface between organically based and functional-mental illnesses was not easy with the Society of Child and Adolescent Psychiatry (Deutsche Gesellschaft für Kinder- und Jugendpsychiatrie DGKJP), officially recognized as specialty since 1968 [4, 24, 26, 27].

**Fig. 5:**
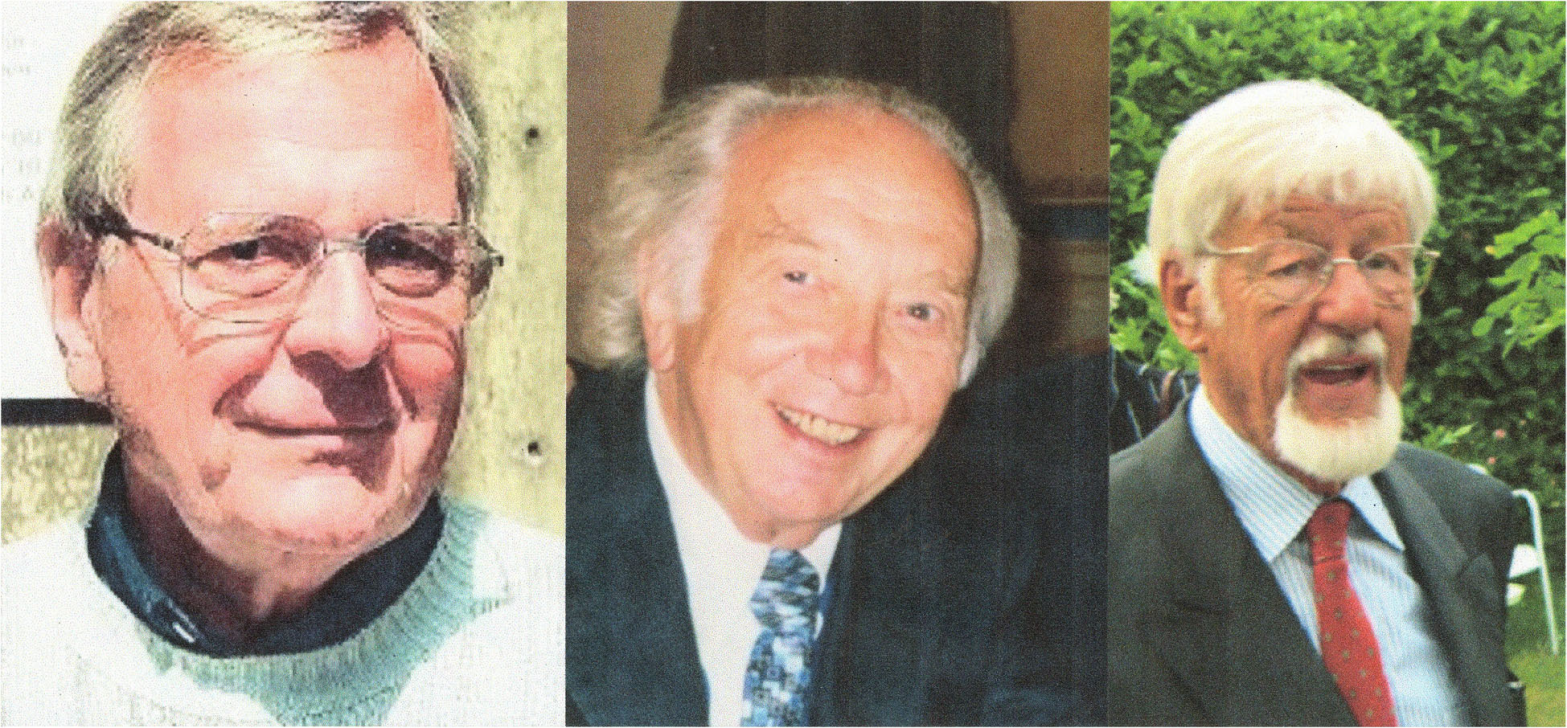
**a**,**b** and **c** Dieter Scheffner, Franz Josef Schulte and Hermann Doose, pioneers of the neuropediatric society GNP in Germany

The Board of the German Society of Pediatrics, but also the dedicated F.J. Schulte initially rejected the establishment of a specialty “child neurology” with the concern this would separate too much from general pediatrics. In contrast, A. Matthes, D. Scheffner and H. Doose advocated the founding of a society for neuropediatrics as a specialty of pediatrics. On June 4th, 1975, the official founding of Gesellschaft für Neuropädiatrie (GNP) took place in Heidelberg with the signatures of H. Doose, D. Scheffner, F. Vasella, R. Kruse, H. Fichsel, H.G. Lenard, J. Martinius and G. Jacobi (Fig. 5).

The first annual meeting of the GNP was organized from November 21st to 23rd 1975 in Heidelberg by D. Scheffner. He defined neuropediatrics as follows: on the one hand it is an integral part of pediatrics and, on the other hand, a link to the neurosciences and complementary to child and adolescent psychiatry. The main topics of the conference were status epilepticus, diagnosis of cognition and neural complication of vaccination.

The second annual meeting of the GNP was successfully organized in 1976 in Kiel by H. Doose, who was supported by the director of the Children’s University Hospital H.R. Wiedemann and his senior physician G. Gross-Selbeck [6]. The Neuropediatric Society was now established as a representation of all pediatricians interested in the diseases of the child’s nervous system.

### Additional activities

Members of the board of the GNP between 1975 and 1990 were among others: D. Scheffner, H. Doose, J. Martinius, H. Fichsel (long-time treasurer), H.G. Lenard (First Secretary), C. Lipinski (Second Secretary), C. Groh, W. Isler, W. Mortier, A. Ritz, B. Ohrt, G. Jacobi, R. Michaelis, G. Neuhäuser, H.M. Weinmann, D.G. Palm, H. Schmutterer, R. Degen and N. Sörensen (as a representative of pediatric neurosurgery). Delegates for the Association of Scientific Medical Societies (AWMF) in Düsseldorf were among others G. Jacobi and H. Fichsel, representatives in the committees of the DGfK G. Neuhäuser, J. Martinius, and F.J. Schulte. As early as 1977, the GNP chairman J. Martinius was in contact with the DGfK chairman H. Ewerbeck with the approval of the entire board because of the additional subspeciality “Neuropediatrics”. However, these applications were not accepted until the 1990s - the unity of pediatrics should be maintained.

An important function had the admission committee: each applicant provided detailed documentation of his neuropediatric activities which were independently assessed by 3 elected members. Prerequisite were e.g. a complete pediatric specialist training, the proof of EEG knowledge, (the so-called “EEG certificate” of the Society of Neurophysiology), a 2 year activity in a department of neuropediatrics or under the guidance of a member of the GNP and, at least in the early years, a 6-month collaboration in a department of adult neurology.

In 1977, the GNP had 141 members, as well as 37 associate members (they were not pediatricians or not predominantly neuropediatricians), in 1986 there were 226 members.

### Further annual meetings

In 1977, the 3rd annual meeting was organized by the neuropediatrician and later child and adolescent psychiatrist J. Martinius of the Max Planck Institute in Munich. The main topics were traumatic brain injury, prevention of children’s accidents, lectures also dealt with urban planning aspects of traffic design. In 1978, Christian Groh organized the conference in Vienna. The conference organized by F. Hanefeld in Berlin in 1979 focused on brain tumors, neurological complications of oncological diseases and treatment-resistant minor seizures. At the 1980 conference in Basel, which was chaired by Werner Isler and Hans Ruedi Hirt, myopathies, cerebrovascular diseases and the neurophysiology of the cerebral cortex were the main topics. For the first time, two-dimensional ultrasound images of the infant’s brain structures were shown and a video recording of the physiotherapeutic treatment of an infant who cried violently during Vojta therapy was vividly discussed [10, 22]. In Frankfurt in 1981, G. Jacobi dealt with the topics of coma, child maltreatment, and oculomotor dysfunctions, 1982 Walter Mortier in Duesseldorf with polyneuropathies and the therapy of neurological diseases. In 1983, R. michaelis and R. Nolte were responsible for the conference in Tübingen, focusing on mental and motor development in the first years of life, developmental neurological diagnostics, treatment of developmental disorders, neurosurgery in the first years of life, neonatal and the febrile convulsions [13]. The Gießen Congress, organized by Gerhard Neuhäuser in 1984, focused on neurogenetics, brain malformations, peri-and postnatal adaptation, and mental retardation [15]. The meeting in Vienna 1985 dealt mainly with psychological influences on the motor system, the neuropediatric patient and his family as well as acute hemisyndromes. At the GNP meeting in 1986 in Bonn, H. Fichsel had selected non-purulent CNS disorders, pediatric migraine, neuroendocrinology, central anticholinergic syndrome and pharmacotherapy of pediatric epilepsy as major themes. Dieter G. Palm arranged the conference 1987 together with E. J. Speckmann, who was responsible for the simultaneous meeting of the League against Epilepsy, in Münster. Epilepsy and neurophysiology, as well as epilepsy surgery, were the topics. 1988 in Munich H.M. Weinmann selected the following main themes: pain, neurocutaneous syndromes, epilepsy, and psychiatric disorders, spinal cord diseases and the investigation of cognitive processes. The annual meeting in 1989 was again organized by F. Hanefeld, now in Göttingen, with the topics progressive encephalopathies, brain tumors, inflammatory CNS diseases and benign epilepsies and in 1990 the topics of neonatal neurology, non-invasive examination methods and seizure disorders were discussed by Joerg Lütschg in Basel.

### Neuropediatric research topics

Some major research projects between 1975 and 1990 were: Epidemiological studies on cerebral palsy - e.g. comparison of German results with Finland and southern Sweden (B. Ohrt, I. Krägeloh-Mann, R. Michaelis), follow-up studies of premature infants in Zurich, Upper Bavarian and Hamburg (R. Michaelis, I. Krägeloh-Mann, B. Ohrt, N Veelken, R. Largo), clinical, EEG and genetic studies on various epilepsies (H. Doose and co-workers, G. Gross-Selbeck, D. Scheffner, R. Kruse, R. Nolte, H.E. Boenigk, H.M. Weinmann and many others), electrophysiology, e.g. evoked potentials (M. Sauer, D. Wenzel, U. Brandl), ultrasound studies using 2-d techniques and Doppler sonography of the CNS (F. Staudt, H.M. Straßburg, K.H. Deeg, H. Bode), cerebral MRI diagnosis (I. Krägeloh-Mann, etc.), CNS inflammations, e.g. Borrelia meningitis and multiple sclerosis (F. Hanefeld, H.J. Christen, J. Gärtner), myology (W. Mortier, R. Beckmann, U. Ketelsen, F. Hanefeld), new syndromes and human genetics, e.g. Rett syndrome (F. Hanefeld, G. Neuhäuser) mitochondriopathies (F. Hanefeld, E. Wilichowski), new metabolic diseases (F. Hanefeld, A. Kohlschütter), pharmacology and new antiepileptic drugs (H.E. Boenigk, H. Doose, G. Gross Selbeck, H. Fichsel, D. Rating), pediatric neurosurgery and epilepsy surgery (N. Sörensen, I. Tuxhorn), brain tumors and oncological diseases (F.J. Schulte, F. Hanefeld, G. Jacobi, R. Korinthenberg, J. Kühl, N. Sörensen), cerebral vascular anomalies (W. Isler, G. Jacobi), child maltreatment (G. Jacobi), neonatal neurology (F.J. Schulte, M. Albani) polygraphy (K.H.P. Bentele, M. Albani), pathophysiology of perinatal brain damage (F.J. Schulte, G. Jorch, H.M. Straßburg), traumatic brain injury in children (R. Korinthenberg, W. Kölfen), etiology of headache and dizziness (M. Förster, H.M. Weinmann), functional disorders, ADHD, psychogenic seizures (J. Martinius), excessive crying in the infant (H.M. Straßburg) and many more [25].

### International contacts

Between 1970 and 1990, in addition to contacts with neuropediatricians in Austria and Switzerland, important contributions were of the Scandinavian countries, England and the USA. Much impact came from the annual meetings of the European Federation of Child Neurology Societies (EFCNS) and their “spiritus rector” Ronny MacKeith, who also founded the journal DEVELOPMENTAL MEDICINE AND CHILD NEUROLOGY and the series CLINICS IN DEVELOPMENTAL MEDICINE (Mac Keith Press). In 1977, EFCNS met in Göttingen and in 1981 in Oxford, official delegates of the GNP were e.g. F. Vasella, F.J. Schulte and F. Hanefeld. Besides, there were meetings every 4 years of the International Child Neurology Association (ICNA), founded in 1972, e.g. 1982 in Copenhagen and 1986 in Jerusalem [1].

Some foreign personalities, to whom good contacts existed, were: USA: A.H. Parmelee, R.C.H. Engel, J. Sinclair, J. Volpe, I. Rapin, D.C. de Vivo, H. Moser, F. Anderman, H.U. Zellweger, J.H. Menkes, S.Di Mauro. GB: MacKeith R., M. Bax, P. Tizard, B., K. Bobath, A. Emery, V. and L. Dubowitz. France: A. Minkowsky, C. Dreyfus-Brissac, J. Aicardi, H. Gastaut, C. Dravet. Italy: A. Milani Comparetti, A. Ferrari, G. Cioni. Scandinavia: B. Hagberg, I. Gamstorp, P. Uvebrandt, H. Lou. Switzerland: G. Dummermuth, N. Herschkowitz. Benelux: H.F.R. Prechtl, B.C.L. Touwen, L. van Bogaert, P. Casaer, J. Willemse. GDR: G. Göllnitz, D. Müller, K.J. Neumarker, H. Todt. CSSR: I. Lesny, V. Vlach Japan: Y. Fukuyama, M. Segawa, etc. [1, 25]

### Discussion about diagnosis and therapy of developmental disorders

**Fig. 6:**
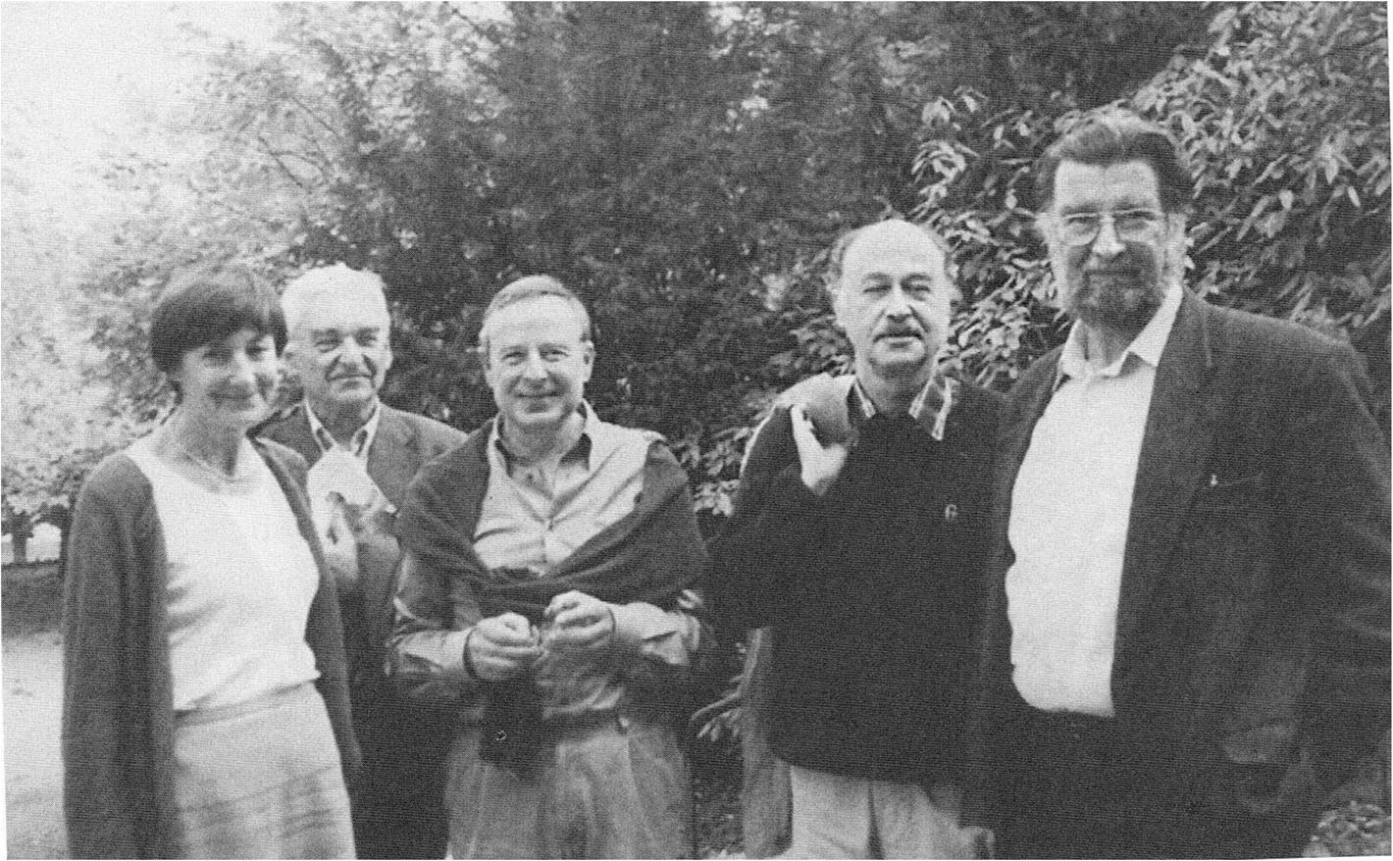
Barbara Ohrt, Richard Michaelis, Hans Georg Schlack, Remo H. Largo und Gerhard Neuhäuser – experts in developmental medicine. Photo of 2002 in Bad Nauheim

Since 1976, GNP has been asked by the German Medical Association to propose criteria for screening examinations introduced since 1971, for which several reports have been submitted. From the end of the 70s onwards, several pediatricians and some physiotherapists, especially Th. Hellbrügge, V. Vojta, H. Bauer, and P. Schulz, promoted a vehement intensification of the earliest physiotherapy in all so-called at-risk children [8]. With the help of the cash-technically billable storage actions a “pre-spastic syndrome” was recognized and the development of cerebral palsy should be prevented by the intensive physiotherapeutic method of reflex locomotion developed by V. Vojta [10, 22, 28]. In the 1980s, the number of “therapy-needy” infants identified by reflexology increased between the U4 and the U6 to 20%, without a decline in the incidence of older children with cerebral palsy in Germany. Even in several follow-up examinations, neurological deficits did not occur in most of these children. Founded in 1982, the GNP developmental neurological working group with Barbara Ohrt, *R. michaelis*, H. G. Schlack, R. H. Largo and G. Neuhäuser worked out criteria for the developmental neurological examination and the resulting therapeutic consequences. They were supported e.g. by Elisabeth Köng from Bern, Heinz F.R. Prechtl and Bert C.L.Touwen from Groningen and Suse Weber from Freiburg [10, 22, 28]. (Fig. 6) Unfortunately, the arguments and allegations used by the other “party” were not always fair, which contributed to the insecurity of many parents and physiotherapists [10]. In 1987 H. Siemes from Berlin applied an opinion on the Doman-Delacato therapy, which i.a. by D. Karch, G. Gross-Selbeck, F. Hanefeld, A. Ritz and H.G. Schlack was taken in a working group, which dealt with complementary therapy methods in neuropediatrics, the results of which were published after 1990.

### Variants of Neuropediatrics and child neurology in Central Europe

In Holland, there were both child neurologists and neuropediaticians until the 90ties. The former came from adult neurology and were specialized in the treatment of diseases such as epilepsy, cerebral palsy, headaches, muscle diseases, etc., the latter being primarily pediatricians dealing with neurological disorders in infants and toddlers and congenital developmental disorders.

In the 50ies Gerhard Göllnitz already had a department for pediatric neuropsychiatry in Rostock in the GDR, his psychologist Bernhard Meyer-Probst established one of the most important longitudinal studies in Germany on developmental prognosis from pregnancy to adulthood. In East-Berlin, Dagobert Müller dealt intensively with aspects of the neurological examination and birth-trauma, Klaus-Jürgen Neumärker with brain stem functions and brain tumors in childhood. The director of the Erfurt Pediatric Clinic Helmut Patzer founded a developmental neurological working group and, together with Roland Eulitz, set up curative education methods for children with developmental disorders. Horst Todt in Dresden dealt intensively with epilepsy and its treatment options [7, 25, 26].

Also, in Austria there was a specialty child neuropsychiatry, which both pediatricians, neurologists, and psychiatrists could acquire if they had also worked for a while in pediatric institutions. A special role played the pediatrician Andreas Rett (1924–1997), who headed a department for behavioral and abnormally developed children at the Vienna-Lainz Hospital and later in the Neurological Clinic at Rosenhügel. In 1966 he first described the syndrome later named after him of a severe general developmental disorder with stereotypic hand movements and reduced head growth, which occurs almost exclusively in girls. The monograph he published in 1971: “Das hirngeschädigte Kind” was considered a standard work for a long time [20]. Because of his advocacy of the sterilization of women with intellectual disabilities and his involvement in the Nazi era, his position today is controversial.

### New points

As early as 1970, R. Kruse, D Scheffner and H.M. Weinmann published a booklet with “Instructions and description of the child’s EEG” [11]. From 1974 this resulted in a working group of neuropediatricians in Königstein, which dealt with topics of epilepsy in children and adolescents e.g. BNS epilepsy, Rolando epilepsy, absence epilepsies, and others among others with H. Doose, H.Fichsel, F. Hanefeld, R. Kruse, C. Lipinski, J. Martinius and Hansjörg Schneble [11]. Doose had also published in 1970 the first edition of the famous monograph “Cerebral seizures in childhood”, which appeared in 11 editions [5].

Meetings of the German League against Epilepsy have been repeatedly organized by several representatives of the GNP. 1990, the conference “The unresolved case” was set up by F. Hanefeld and his co-workers in Göttingen.

Departments with a focus on neuropediatrics at Children’s University Hospitals were first established in Göttingen, later in Berlin, Gießen, Heidelberg, Kiel, and Tübingen. A Department of Childhood Muscle Diseases, founded in 1975 in Freiburg with Robert Beckmann, Kai Uwe Ketelsen and Manfred Sauer promoted the foundation of the German Society of Muscular Diseases (DGM).

Since the end of the 1980s, social pediatric centers (SPZ - Sozialpädiatrische Zentren) have increasingly been opened in Germany, following the example of the Children’s Center in Munich, which was founded in 1968 by Theodor Hellbrügge. An increasing number of children with chronic CNS disorders and multiple disabilities were treated interdisciplinarily with psychologists, therapists, special education and music therapy [8].

The introduction of the improvement of neuropediatric quality has been discussed since 1976. F.J. Schulte reports that in the early years he repeatedly pointed out that it was necessary to define exactly what a neuropediatrician should know and should be able to do. During a lecture in the circle of Ronny MacKeith he used the word “training” again and again. Afterward, Peter Tizard spoke with all the elitist power of his Oxford-trained language: “Dr. Schulte, in the United Kingdom, training is for dogs, soldiers, and surgeons. Neuropediatricians should be educated! ”.

## Conclusions

Since the first annual meeting of the GNP in 1975, the Neuropediatric Society had developed continuously and at a high scientific level. Its competence as an important specialty for all neurological diseases in children and adolescents in German-speaking countries was no longer questioned. Many diseases were more clearly defined by new methods, in particular, X-ray and magnetic resonance computed tomography and ultrasound imaging and electrophysiology, which had important implications for explanations of pathophysiology and, in part, for therapy. The fact that much more knowledge should be possible using the nuclear spin technique and the investigation possibilities of genetics was not fully recognizable until 1990. With the advocacy of other medical societies, especially child and adolescent psychiatrists, adult neurologists, social pediatricians, pediatric oncologists, neonatologists, pediatric orthopedists and physiotherapists and occupational therapists, there were always discussions about the responsibility and thus about the decision-making authority and billing options, e.g. in neuropsychological disorders such as ADHD and tics, follow-up of risk infants, muscle diseases, brain tumors and cerebral palsy, and treatment indications for children with developmental problems. For the established pediatricians were the topics of the GNP partially too academic, the influential Th. Hellbruegge repeatedly stated that the neuropediatricians “would not adequately consider the concerns of the practice. It is more important to initiate therapy for developmental abnormalities than to carry out extensive and burdensome examinations “ [8]. At the larger children’s hospitals and increasingly in the new social pediatric centers newly founded since the mid-1980s, members of the GNP were in great demand. On the one hand, they were needed for the large number of patients with diseases of the nervous system that were treated as in- and outpatient and, on the other hand, they made connections to basic sciences, e.g. in the field of biochemistry, neurophysiology, neuropsychology, neuroradiology, and human genetics. Thus the demand became ever louder to introduce a recognition for the emphasis Neuropaediatrics, which was possible however only in the context of a comprehensive change of a revised medical education order, which was finally implemented in 2004.

## Data Availability

Not applicable.

## Abbreviations

ADHD: Attention Deficit Hyperactivity Disease
DGfK: Deutsche Gesellschaft für Kinderheilkunde
DGKJP: Deutsche Gesellschaft für Kinder- und Jugendpsychiatrie
DVJ: Deutsche Vereinigung für Jugendpsychiatrie
EFCNS: European Federation of Child Neurology Societies
GDR: German Democratic Republic
GNP: Gesellschaft für Neuropädiatrie
ICNA: International Child Neurology Association
ILAE: International League Against Epilepsy
NSDAP: Nationalsozialistische Partei Deutschland
U4/U6: 4th and 6th preventive medical check-up
